# Supplementary material for: Leading basic modes of spontaneous activity drive individual functional connectivity organization in the resting human brain
Source: Commun Biol. 2023 Aug 31;6:892. doi: 10.1038/s42003-023-05262-7 (PMC10471630; doi:10.1038/s42003-023-05262-7)
Supplement: Supplementary file 3 — Reporting Summary [file 42003_2023_5262_MOESM3_ESM.pdf]

Corresponding author(s): Xuhong Liao
Last updated by author(s): Aug 16, 2023

Reporting Summary

Nature Portfolio wishes to improve the reproducibility of the work that we publish. This form provides structure for consistency and transparency in reporting. For further information on Nature Portfolio policies, see our Editorial Policies and the Editorial Policy Checklist.

Statistics

For all statistical analyses, confirm that the following items are present in the figure legend, table legend, main text, or Methods section.

n/a	Confirmed	
☐	☒	The exact sample size (*n*) for each experimental group/condition, given as a discrete number and unit of measurement
☐	☒	A statement on whether measurements were taken from distinct samples or whether the same sample was measured repeatedly
☐	☒	The statistical test(s) used AND whether they are one- or two-sided<br>*Only common tests should be described solely by name; describe more complex techniques in the Methods section.*
☒	☐	A description of all covariates tested
☐	☒	A description of any assumptions or corrections, such as tests of normality and adjustment for multiple comparisons
☐	☒	A full description of the statistical parameters including central tendency (e.g. means) or other basic estimates (e.g. regression coefficient) AND variation (e.g. standard deviation) or associated estimates of uncertainty (e.g. confidence intervals)
☐	☒	For null hypothesis testing, the test statistic (e.g. F, t, r) with confidence intervals, effect sizes, degrees of freedom and P value noted<br>*Give P values as exact values whenever suitable.*
☒	☐	For Bayesian analysis, information on the choice of priors and Markov chain Monte Carlo settings
☒	☐	For hierarchical and complex designs, identification of the appropriate level for tests and full reporting of outcomes
☐	☒	Estimates of effect sizes (e.g. Cohen's *d*, Pearson's *r*), indicating how they were calculated

Our web collection on statistics for biologists contains articles on many of the points above.

Software and code

Policy information about availability of computer code

Data collection	No software was used to collect data.
Data analysis	The MATLAB toolbox GRETNA (Wang et al., 2015) (https://www.nitrc.org/projects/gretna/) was used to preprocess the functional data.<br>The MATLAB toolbox BrainNet Viewer (Xia et al., 2013) (https://www.nitrc.org/projects/bnv/) was used to visualize brain maps.<br>The Python package kneed (https://github.com/arvkevi/kneed) was used to identify the elbow point on the discrete curve.<br>The codes used to perform the analysis are freely available at https://github.com/liaolab-bnu/LeadingModes_rfMRI.

For manuscripts utilizing custom algorithms or software that are central to the research but not yet described in published literature, software must be made available to editors and reviewers. We strongly encourage code deposition in a community repository (e.g. GitHub). See the Nature Portfolio guidelines for submitting code & software for further information.

Data

Policy information about availability of data

All manuscripts must include a data availability statement. This statement should provide the following information, where applicable:
- Accession codes, unique identifiers, or web links for publicly available datasets
- A description of any restrictions on data availability
- For clinical datasets or third party data, please ensure that the statement adheres to our policy

Three R-fMRI datasets were used in the current study. The S900 release of the Human Connectome Project (HCP) dataset is publicly available at https://

www.humanconnectome.org/study/hcp-young-adult/data-releases. The Beijing Zang dataset is publicly available at https://www.nitrc.org/projects/fcon_1000. The sleep-deprivation dataset is available upon reasonable request. Maps of leading basic modes and some other data supporting our results are available at https://github.com/liaolab-bnu/LeadingModes_rfMRI.

Research involving human participants, their data, or biological material

Policy information about studies with human participants or human data. See also policy information about sex, gender (identity/presentation), and sexual orientation and race, ethnicity and racism.

Reporting on sex and gender	For the HCP dataset, 700 participants (M/F: 304/396) were used. For the sleep-deprivation dataset, 19 participants (M/F:7/12) were used. For the Beijing Zang dataset, 197 participants (M/F: 75/122) were used. The sex effect was not examined.
Reporting on race, ethnicity, or other socially relevant groupings	n/a
Population characteristics	For the HCP dataset, 700 participants aged 21-35 years. For the sleep-deprivation dataset, 19 participants aged 18-26 years. For the Beijing Zang dataset, 197 participants aged 18-26 years. All participants had no history of neurological and psychiatric disorders.
Recruitment	For the HCP dataset, R-fMRI data were selected from the S900 release of the HCP project (https://www.humanconnectome.org/study/hcp-young-adult/data-releases). For the Beijing Zang dataset, R-fMRI data were obtained from the 1000 Funcitonal Connectomes Project (FCP) (http://www.nitrc.org/projects/fcon_1000/). For the sleep-deprivation dataset, volunteers were recruited from respondents to a web-based questionnaire (Zhou et al., 2022).
Ethics oversight	For the HCP dataset, the scanning protocol was approved by the Institutional Review Board at Washington University. For the sleep-deprivation dataset, the research was approved by the Institutional Review Board of the Institute of Biophysics (Chinese Academy of Sciences). For the Beijing Zang dataset, the research was approved by the Institutional Review Board of the State Key Laboratory of Cognitive Neuroscience and Learning at Beijing Normal University.

Note that full information on the approval of the study protocol must also be provided in the manuscript.

Field-specific reporting

Please select the one below that is the best fit for your research. If you are not sure, read the appropriate sections before making your selection.

☒ Life sciences ☐ Behavioural & social sciences ☐ Ecological, evolutionary & environmental sciences

For a reference copy of the document with all sections, see nature.com/documents/nr-reporting-summary-flat.pdf

Life sciences study design

All studies must disclose on these points even when the disclosure is negative.

Sample size	Initial sample sizes: N=837 for the HCP dataset; N=20 for the sleep-deprivation dataset; N=198 for the Beijing Zang dataset.
Data exclusions	For the HCP dataset, 137 participants were excluded due to missing time points (N=10), excessive head motion (N=105) and arachnoid cysts (N=22). For the sleep-deprivation dataset, one participant was excluded due to excessive head motion. For the Beijing Zang dataset, 1 participant was excluded due to differences in scanning orientation.
Replication	Five analysis strategies were considered to verify the reproducibility , including (i) varying the number of participants; (ii) using stricter head motion exclusion criteria; (iii) performing nuisance regression without global signal regression; (iv) defining brain nodes based on two functional parcellations with different spatial resolutions; and (v) using another independent dataset, i.e., the Beijing Zang dataset. The number and spatial patterns of the leading basis modes were examined in these cases.
Randomization	n/a
Blinding	n/a

Reporting for specific materials, systems and methods

We require information from authors about some types of materials, experimental systems and methods used in many studies. Here, indicate whether each material, system or method listed is relevant to your study. If you are not sure if a list item applies to your research, read the appropriate section before selecting a response.

Materials & experimental systems

n/a	Involved in the study
☒	☐ Antibodies
☒	☐ Eukaryotic cell lines
☒	☐ Palaeontology and archaeology
☒	☐ Animals and other organisms
☒	☐ Clinical data
☒	☐ Dual use research of concern
☒	☐ Plants

Methods

n/a	Involved in the study
☒	☐ ChIP-seq
☒	☐ Flow cytometry
☐	☒ MRI-based neuroimaging

Magnetic resonance imaging

Experimental design

Design type: Resting-state functional MRI

Design specifications: n/a

Behavioral performance measures: n/a

Acquisition

Imaging type(s): Resting-state functional MRI

Field strength: 3T

Sequence & imaging parameters: For the HCP dataset, R-fMRI scans were obtained using a multiband gradient echo-planar imaging sequence with the following parameters: repetition time = 720 ms, echo time = 33.1 ms, flip angle = 52 degree, bandwidth = 2290 Hz/pixel, field of view = 208 mm*180 mm, matrix = 104*90, 72 slices, 2.0 mm isotropic voxels, multiband acceleration factor = 8, and 1200 volumes (14 min and 24 s) for each run.
For the sleep-deprivation dataset, R-fMRI scans were obtained using a multiband gradient echo-planar imaging sequence with the following parameters: repetition time = 1000 ms, echo time = 29 ms, flip angle = 40 degree, field of view = 192 mm*192 mm, matrix = 96*96, 65 slices, slice thickness/gap = 2.0/0 mm, multiband acceleration factor = 5, and 480 volumes (8 min) for each run.
For the Beijing Zang dataset, R-fMRI scans were obtained using a gradient echo-planar imaging sequence with the following parameters: repetition time = 2000 ms, echo time = 30 ms, flip angle = 90 degree, field of view = 200 mm * 200 mm, matrix = 64*64, 33 slices, slice thickness/gap = 3/0.6 mm, and 235 volumes (7 min and 50 s) for each run.

Area of acquisition: whole brain

Diffusion MRI: ☐ Used ☒ Not used

Preprocessing

Preprocessing software: GRENTA (Wang et al., 2015) (https://www.nitrc.org/projects/gretna/)

Normalization: For the HCP dataset, we employed the minimally preprocessed R-fMRI data (Glasser et al., 2013), which have been projected to the Montreal Neurological Institute (MNI) space (Glasser et al., 2013).
For the sleep-deprivation dataset and the Beijing Zang dataset, R-fMRI data were spatially normalized to the MNI space with the T1-unified segmentation algorithm (Ashburner and Friston, 2005).

Normalization template: MNI152

Noise and artifact removal: For the HCP dataset, we employed the minimally preprocessed R-fMRI data (Glasser et al., 2013), followed by ICA-Fix denoising (Griffanti et al., 2014). Four additional steps were performed during the preprocessing, including the removal of the first 10-second volumes, linear detrending, nuisance regression, and temporal filtering (0.01-0.08Hz). During the nuisance regression, whiter matter, cerebrospinal fluid, and global brain signals were included as regressors to further remove the influence of head motion and physiological noise.
For the sleep-deprivation and the Beijing Zang dataset, the preprocessing steps included the removal of the first 10-second volumes, realignment, spatial normalization, linear detrending, nuisance regression, and temporal filtering (0.01-0.08Hz). During the nuisance regression, we included Friston's 24 head-motion parameters, white matter, cerebrospinal fluid, and global brain signals as regressors to reduce the influence of head motion and physiological noise.

Volume censoring: n/a

Statistical modeling & inference

Model type and settings: For the sleep-deprivation dataset, we performed a permutation test (n = 10,000) to estimate the effect of sleep deprivation.

Effect(s) tested: sleep-deprivation

Specify type of analysis: ☐ Whole brain ☒ ROI-based ☐ Both

Anatomical location(s): ROIs were defined based on a prior functional parcellation (Schaefer et al., 2018).

Statistic type for inference (See Eklund et al. 2016): We performed statistical analyses at both the ROI-wise and system levels with correction for multiple comparisons.

Correction: Multiple comparisons were corrected using the false discovery rate method, with corrected $p < 0.05$.

Models & analysis

n/a	Involved in the study
☐	☒ Functional and/or effective connectivity
☒	☐ Graph analysis
☐	☒ Multivariate modeling or predictive analysis

Functional and/or effective connectivity: We used Pearson's correlation to estimate functional connectivity.

Multivariate modeling and predictive analysis: Eigen-microstate analysis from the statistical physics theory was applied to regional R-fMRI time courses.
